# Effects of choline containing phospholipids on the neurovascular unit: A review

**DOI:** 10.3389/fncel.2022.988759

**Published:** 2022-09-23

**Authors:** Proshanta Roy, Daniele Tomassoni, Giulio Nittari, Enea Traini, Francesco Amenta

**Affiliations:** ^1^School of Biosciences and Veterinary Medicine, University of Camerino, Camerino, Italy; ^2^School of Medicinal and Health Products Sciences, University of Camerino, Camerino, Italy

**Keywords:** neurovascular unit, choline containing phospholipids, phosphatidylcholine, CDP-choline, choline alphoscerate

## Abstract

The roles of choline and of choline-containing phospholipids (CCPLs) on the maintenance and progress of neurovascular unit (NVU) integrity are analyzed. NVU is composed of neurons, glial and vascular cells ensuring the correct homeostasis of the blood-brain barrier (BBB) and indirectly the function of the central nervous system. The CCPLs phosphatidylcholine (lecithin), cytidine 5′-diphosphocholine (CDP-choline), choline alphoscerate or α-glyceryl-phosphorylcholine (α-GPC) contribute to the modulation of the physiology of the NVU cells. A loss of CCPLs contributes to the development of neurodegenerative diseases such as Alzheimer’s disease, multiple sclerosis, Parkinson’s disease. Our study has characterized the cellular components of the NVU and has reviewed the effect of lecithin, of CDP-choline and α-GPC documented in preclinical studies and in limited clinical trials on these compounds. The interesting results obtained with some CCPLs, in particular with α-GPC, probably would justify reconsideration of the most promising molecules in larger attentively controlled studies. This can also contribute to better define the role of the NVU in the pathophysiology of brain disorders characterized by vascular impairment.

## Introduction

**FIGURE 1:**
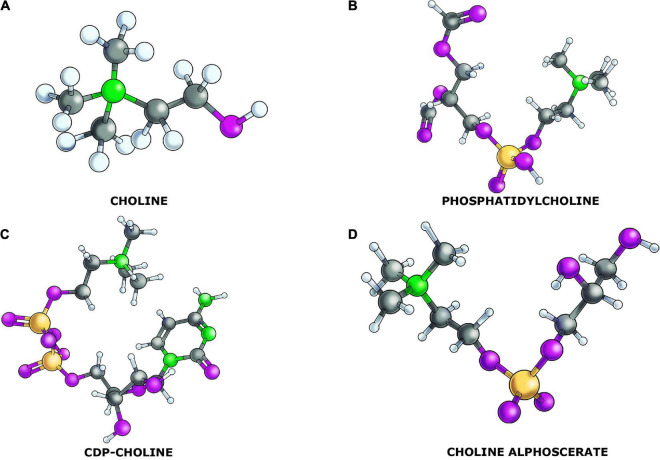
Chemical structure of the choline containing phospholipids (CCPLs) and the precursor choline: **(A)** choline; **(B)** phosphatidylcholine; **(C)** CDP-Choline; and **(D)** choline alphoscerate.

Choline plays a prominent role in the synthesis of different membrane phospholipids (Javaid et al., 2021). Moreover, choline is essential for cholinergic neurons to synthesize the neurotransmitter acetylcholine. The compound *via* choline-containing phospholipids (CCPLs, Figure 1) modulates various pathways of intercellular communication and pathophysiological processes through neurovascular coupling (Attwell et al., 2010), cortical spreading depression (Gursoy-Ozdemir et al., 2004), or induction of oxidative stress (Yemisci et al., 2009). Choline is required for the synthesis of phosphatidylcholine, lyso-phosphatidylcholine, sphingomyelin, and choline plasmalogen, as well as other phospholipids. It has a well-established involvement in neurogenesis and memory development, and its absence may result in neural tube abnormalities (Paoletti et al., 2011). CCPLs such as phosphatidylcholine or lecithin, cytidine 5′-diphosphocholine (CDP-choline) and alpha-glyceryl-phosphorylcholine or choline alphoscerate (α-GPC) are acetylcholine (ACh) precursors that easily traverse the blood brain barrier (BBB). They act through two separate mechanisms of action. The precursors are first used as a substrate for acetylcholine synthesis, which can improve cholinergic neurotransmission (Hurtado et al., 2011). ACh precursors have been shown to prolong the favorable effects of acetylcholinesterase inhibitors on cognitive and behavioral improvements in patients with Alzheimer’s disease (AD) and mild to moderate vascular dementia (Adibhatla et al., 2005; Amenta et al., 2020). They may have significant neuroprotective benefits in addition to serving as precursors by maintaining and supplying the membrane structure of neurons (Rao et al., 2000). Previous research has shown that precursor administration to rats 3 weeks after a pilocarpine-induced seizure increased neurogenesis. This suggests that ACh precursors can be used not only as neuroprotective substances but also to aid neurogenesis after ischemic or epileptic damage (Diederich et al., 2012; Lee et al., 2017). CCPLs and the acetylcholinesterase inhibitors were the treatments more largely studied in clinical trials for the AD consistent with the hypothesis that a replacement/enhancement of the cholinergic function may be useful in the treatment of AD patients. Based on the available results, it has been hypothesized that CCPLs could still have a place in the pharmacotherapy of adult-onset dementia disorders (Amenta et al., 2020).

CCPLs participate in the biosynthesis of the cell membrane phospholipids. Phosphatidylcholine is the main form of phosphoglycerides that contains the choline molecule as the head group. It represents the main phospholipid of the outer layer of the cellular and intracellular membranes of mammalian cells, and it accounts for 32.8% of the total glycerophospholipid content of the human brain (Javaid et al., 2021). The synthesis could be mediated by the direct methylation of the ethanolamine residue of phosphatidylethanolamine or *via* the Kennedy pathway. Choline molecules are phosphorylated by choline kinases, which after processing by cytidylyltransferase, generated CDP-choline, which further couples with phosphatidic acid and gives phosphatidylcholine (Javaid et al., 2021). Other CCPLs such as sphingomyelin, are abundant in the myelin sheath and contribute to maintain the integrity of the axonal covering (Javaid et al., 2021).

### The neurovascular unit

**FIGURE 2:**
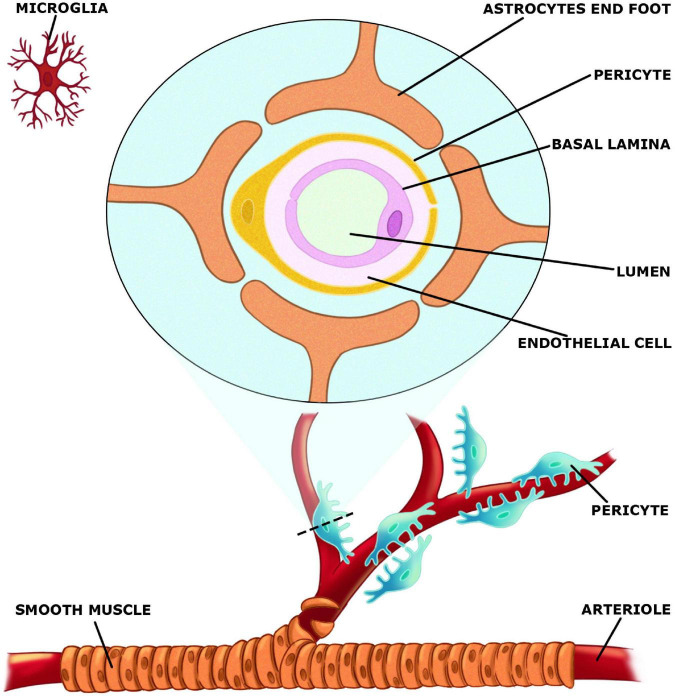
Elements of the neurovascular unit (NVU).

The neurovascular unit (NVU) (Figure 2), which is made up of both neurons, glial and vascular cells is responsible for integrating changes in blood supply to increases or decreases in neuronal activity (Kim and Filosa, 2012). Vascular components of NVU include endothelial cells, pericytes, and vascular smooth muscle cells, whereas glial cells include astrocytes, microglia, and oligodendroglia. Acetylcholine as well as CCPLs play an active role in the regulation of NVU. These molecules display protective effects on neuronal death, microglial activation, BBB disruption and consequent neurological injury (Inazu, 2019). NVU is a relevant structure in the maintenance of brain homeostasis. The concept of NVU was introduced Harder et al. (2002) as a brain structure formed by neurons, interneurons, astrocytes, basal lamina covered with smooth muscular cells and pericytes, endothelial cells and extracellular matrix (Muoio et al., 2014). Each element is intimately and reciprocally interconnected, establishing a comprehensive anatomical and functional system of cerebral blood flow control (Abbott and Friedman, 2012).

In the central nervous system (CNS), astrocytes are the most common glial cells. Astrocytes are essential for molecular transport and the integrity of the BBB and through their end-feet, astrocytes connect with endothelial cells in the NVU (Yamazaki and Kanekiyo, 2017). Neurovascular coupling is a process in which astrocytes link neural activity with blood arteries. They react to neural activity and send out signals to control cerebral blood flow (CBF) (Gordon et al., 2011). Astrocytes are formed in the last stages of neurogenesis, along with neurons and oligodendrocytes. As multifunctional cells, astrocytes are strongly connected with neurons and blood vessels, and communicate with neuronal pre-and post-synaptic terminals to help control synaptic transmission by releasing glutamate, D-serine, and ATP (López-Bayghen and Ortega, 2011; Santello et al., 2012). Astrocytes can be largely coupled into syncytial structures of up to 100 units by gap junctions. Extend end-feet process can modulate CBF or the BBB and high levels of aquaporin-4 water channel proteins enhance perivascular clearance by the newly identified “glymphatic system” (Plog and Nedergaard, 2018).

Microglia cells are derived from the yolk sac and seed in the brain as the first glial cells. During development they grow as highly flexible cells with mobility alongside neurons (Pont-Lezica et al., 2011). Through their interaction in the NVU, active microglia and astrocytes may reach a condition of immunological “optimization”. Microglia cells, which are found throughout the brain, differentiate early in embryonic development or as the first response of the CNS to neuroinflammation (Klein and Hunter, 2017).

In the brain, endothelial cells constitute the tubes represented by capillaries. The BBB is formed of continuous endothelial cells with tight junctions, basement membrane, and astrocyte end-feet in the NVU. BBB controls through chemical and/or anatomic barriers substances entering the brain, and removes dangerous proteins from the brain parenchyma into the bloodstream (Yamazaki and Kanekiyo, 2017). Endothelial cells are joint by tight junctions and adherent junctions, and tight junctions reduce BBB paracellular permeability. All the elements, when combined with neurons, constitute the NVU (Zlokovic, 2008).

The NVU maintains the optimal functioning of the brain microenvironment, contributing to neuronal survival, and information processing by regulating BBB permeability and CBF. Vascular dysfunction has been implicated in various neurodegenerative disorders. The NVU may be damaged, causing BBB malfunction and a drop in CBF, which may concur to the pathophysiology of neurodegenerative diseases. When the NVU is desegregated and CBF drops, the supply of oxygen and nutrients to the brain is reduced as well as the clearance of neurotoxic substances (Sweeney et al., 2018). Claudins, that occlude junctional adhesion molecule, and zonula occludens-1 are all transmembrane proteins involved in the formation of tight junctions (Yamazaki and Kanekiyo, 2017; Bell et al., 2010). The main cadherin that forms the adherent’s junction and mediates intercellular adhesion is vascular endothelial cadherin (Zenaro et al., 2017). Tight and adherent junctions are critical in controlling endothelial permeability and help to support normal brain physiological function by limiting the entry of macromolecules, toxins, wastes and perilous blood-borne pathogens (Daneman and Prat, 2015). Tight junctions restrict protein diffusion and seal the paracellular cleft between endothelial cells, whereas adherents’ junctions are important for cell-to-cell interaction and cell growth (Tietz and Engelhardt, 2015).

Pericytes cover the abluminal surface of capillaries and adjust capillary diameter to regulate blood flow. BBB permeability, CBF, immunological trafficking, trans endothelial fluid transport, and vascular integrity are all regulated by pericytes (Brown et al., 2019). Pericytes are also involved in the establishment of tight junctions and remove harmful proteins to keep the CNS stable (Daneman et al., 2010; Sagare et al., 2013). Furthermore, for the development, maintenance, and effective functioning of the BBB, interaction between endothelial cells and pericytes is essential (Geranmayeh et al., 2019). Table 1 summarizes the various component of the NVU.

**TABLE 1:** Various components that constitute the neurovascular unit.

NVU components	Classification	Receptors and subtypes	NVU antibodies/Markers
Microglia	Ramified or dormant microglia	Complement type 3 receptor (CR3)	OX-42, MAC-1, CD11b/CD18, OX-6, OX-6, OX-17, and OX-3
	Activated microglia	Major histocompatibility complex class II (MHC II)	CD68, clone ED1, Iba1
	Phagocytic or amoeboid	Major histocompatibility complex class I (MHCI), CD4 receptor	OX42, F4/80, ED1 and ED2, OX-18, OX-6, OX-17, OX-1, and OX-35
	Perivascular microglia	Major histocompatibility complex class II (MHC II)	Iba-1, ED-1, ED-2, OX-17, OX-18 and F4/80, and OX-42
Astrocytes			Glial fibrillary acidic protein (GFAP)
Neurons	Mature neurons	*N*-methyl-D-aspartate (NMDA) 1 glutamate receptors	Neuronal antigen nuclei (NeuN), HuC/D RNA-binding proteins
Pericytes		The tyrosine-kinase receptor PDGFRβ	Platelet-derived growth factor receptor β (PDGFRβ)
			α-Smooth muscle actin (α-SMA)
			Neuron-glial 2 (NG2)
			Desmin (cytoskeleton)
Endothelial cells	Tight junctions proteins	Claudins	Claudin-1, Claudin-3, Claudin-5, and Claudin-12
		Occludins	Zonula occludens (ZO)-1 and ZO-2, ZO-3
		Junctional adhesion molecule (JAM)	VE-cadherin, β-catenin, Caveolin-1, plasma lemma vesicle-associated protein (PLVAP), Platelet-endothelial cell adhesion molecule (PECAM-1), Intercellular adhesion molecule 1 (ICAM-1), Vascular endothelial cell adhesion molecule-1 (VECAM-1)
Basement membrane			Bulins1 (or BM90) and 2; thrombospondins 1 and 2, BM40, Laminin, Collagen IV, Agrin, and Fibronectin
Other blood brain barrier marker		Aquaporins (AQPs)	AQP1, AQP4, and AQP9
		β-Dystroglycan	Dystrophin-glycoprotein complex (DGC), and laminin 2
		Matrix metalloproteinases (MMPs)	Collagenases (MMP-1, MMP 8, MMP-13, MMP-18), gelatinases (MMP-2, MMP-9), stromelysins (MMP-3, MMP-10, MMP-11), matrilysins (MMP-7, MMP-26) and membrane type MMPs (MMP-14, MMP-15, MMP-16, MMP-17, MMP-24, and MMP-25)

## Choline containing phospholipids

### Phosphatidylcholine or lecithin

Neuronal membranes contain different types of lipids present in different amounts: glycerolipids (60%), sterols (20%), glycosphingolipids (15%), and sphingomyelin (5%). Phosphatidylcholine is the most prevalent phospholipid in mammalian membranes, accounting for the 58% of all phospholipids. Other lipids, like sphingolipids and sterols, play key functions during neuronal differentiation (Vance et al., 2000; Schnaar, 2010).

Phosphatidylcholine is a choline-containing, zwitterionic phospholipid (Figure 1B). The quaternary amine choline head group of phosphatidylcholine cannot be substituted by primary or secondary amine analogues without adversely affecting cell physiology. Due to its abundance in mammalian cell membranes, depriving cells of phosphatidylcholine by inhibiting synthesis or availability of the precursors results in growth arrest and apoptosis (Ridgway, 2021).

Local synthesis produces half of the phosphatidylcholine that accumulates in distal axons, whereas the other half comes through transport from nerve cell bodies and proximal axons. It is well known that phosphatidylcholine biosynthesis, as a major phospholipid of mammalian membrane, increases during neurogenesis (Carter et al., 2003; Gil et al., 2004). The way through which phosphatidylcholine production is adequately increased to continue growing, as well as the signals that coordinate these processes, are poorly understood. Some evidence demonstrated that phosphatidylcholine or its metabolites could act as a neurotrophic-like factor modulating cell fate and neuronal plasticity (Marcucci et al., 2010). Phosphatidylcholine is the most abundant phospholipid in mammalian plasma and intracellular membranes. Phosphatidylcholine is synthesized from choline through the CDP-choline or Kennedy pathway. In a reaction catalyzed by phosphatidylethanolamine *N*-methyltransferase, it can also be made by directly methylating the ethanolamine residue of phosphatidylethanolamine to form phosphatidylcholine (Vance and Vance, 2004). Phosphatidylcholine-specific phospholipase C activation and phosphocholine cytidylyl-transferase down-regulation inducing accumulation of phosphatidylcholine in membranes, leading to an increase in cell viability (Adibhatla and Hatcher, 2005).

### Cytidine 5′-diphosphocholine or citicoline

Cytidine 5′-diphosphocholine, CDP-choline or citicoline, is composed of cytidine and choline linked by a diphosphate bridge (Figure 1C). It is an intermediary in the biosynthesis of cellular membrane phospholipids and is particularly relevant in the production of phosphatidylcholine (Kim J. H. et al., 2015).

From a pharmacological point a view, CDP is classified as a nootropic agent, e.g., a drug used to specifically improve learning or memory, particularly to prevent the cognitive decline associated with dementias (Fioravanti and Yanagi, 2005). Studies in growing sympathetic neurons obtained from rat superior cervical ganglia, showed that when the intracellular concentration of choline is reduced, CDP-choline has beneficial effects in several CNS injury models and pathological conditions. These include cerebral ischemia, traumatic brain injury, hypoxia, AD and Parkinson’s diseases. It was proposed that CDP-choline exerted its beneficial effect by attenuating ischemia-induced phospholipase A2.

CDP-choline supplementation promotes brain metabolism by enhancing the synthesis of acetylcholine, and elevates noradrenaline, dopamine, and serotonin levels. CDP-choline restoring neuronal phospholipid affects neuronal membrane excitability and increases neurotransmitter concentrations. The main products of CDP-choline pathways (1,2-diacyl-glycerophosphocholine; sphingomyelin glycerophosphocholine) are incorporated into the membrane phospholipid structure, improving metabolism of mitochondria and phospholipid synthesis (Fagone and Jackowski, 2013).

When administered orally or intravenously, CDP-choline releases its two principal components, cytidine and choline. CDP-choline is virtually entirely absorbed when taken orally, and its bioavailability is the same as when taken intravenously. It has been reported that and citicoline could reverse or prevent neuronal injury (Adibhatla and Hatcher, 2005). Cytidine and choline are absorbed and dispersed throughout the body, crossing the BBB on their way to the CNS, where they are integrated into the phospholipids component of the membrane and microsomes (Secades and Frontera, 1995). In the CNS, citicoline has been demonstrated to increase ACh, noradrenaline, and dopamine levels (Secades and Lorenzo, 2006). In view of its membrane-stabilizing effects, citicoline may affect the BBB integrity (Rao et al., 2000). CDP-choline boosts brain metabolism and affects the levels of several neurotransmitters by activating structural phospholipids in neuronal membranes. Moreover, CDP-choline has been demonstrated to increase dopamine and noradrenaline levels in the CNS in an experimental setting. CDP-choline has a neuroprotective impact in ischemia and hypoxia improving learning and memory function in animal models of brain aging, owing to these pharmacological properties (Tayebati et al., 2011).

### α Glyceryl-phosphorylcholine or choline alphoscerate

α-GPC is semi-synthetic derivative of lecithin. It is a member of the class of CCPLs composed by the choline ester of sn-glycero-3-phosphate (Figure 1D). It is also a parasympathomimetic ACh precursor which has been investigated for phosphorylcholine the treatment of AD and other forms of dementia.

α-GPC after administration, is transformed into the metabolically active form of choline, phosphoryl choline that reaches the cholinergic nerve terminals and stimulates ACh synthesis (Amenta et al., 2001). α -GPC improves learning and memory in animal models by increasing acetylcholine levels in the hippocampus (Traini et al., 2013). The cognitive activity of the compound was also demonstrated on symptoms of AD in clinical settings (De Jesus Moreno, 2003). Studies conducted on animals suffering from seizures have suggested that cognitive improvement may depend on increased neuroblast formation, reduced neuronal death, and BBB disruption. Based on these results it has been hypothesized that the compound may contribute to improve cognition in epileptic patients (Lee et al., 2017).

α-GPC is involved in the formation of nerve cell membranes. It is a source of choline that is rapidly absorbed, with the advantage of not carrying the electrical charge of native choline (Parnetti et al., 2001). This probably allows an easier crossing of the BBB. Preclinical studies have shown that α -GPC increases the release of acetylcholine in the rat hippocampus, promotes learning and memory and counters cognitive impairment in the experimental brain aging model of scopolamine-induced memory deficits. Moreover, it counters micro anatomical brain alterations and impairment of cholinergic neurotransmission markers and receptors in old rats (Parnetti et al., 2007).

α-GPC interferes with brain phospholipid metabolism and increases brain choline and ACh synthesis and release. Pharmacodynamic studies on α -GPC during phases of development of the compound were focused primarily on its role in improving brain cholinergic neurotransmission and in interfering with brain phospholipid metabolism. Preclinical studies have demonstrated that α -GPC increases the release of ACh in rat hippocampus, facilitates learning and memory in experimental animals, improves brain transduction mechanisms and decreases the age-dependent structural alterations occurring in the rat brain areas such as frontal cortex and hippocampus. Furthermore, α -GPC contributes to anabolic processes responsible for membrane phospholipid and glycerol-lipid synthesis, positively influencing membrane fluidity. Based on the evidence, the central parasympathomimetic activity of the compound was defined, suggesting its clinical use in patients with cognitive decline (Traini et al., 2013).

## Role of choline-containing phospholipids on neurovascular unit elements

### Choline-containing phospholipids effects on neurons

Neuroinflammatory response, oxidative stress, protein homeostasis, and apoptotic signaling are involved in the cognitive decline and in the development of neurodegenerative disorders (Teleanu et al., 2022). CDP-choline has been used as a therapeutic agent in combination with levodopa in the treatment of Parkinson’s disease (PD). Citicoline reduces the cytotoxic effect of 6-hydroxydopamine (6-OHDA)-treated human dopaminergic SH-SY5Y neuroblastoma cells. Moreover, CDP-choline significantly attenuates substantia nigra dopaminergic cell dropout and tyrosine hydroxylase immunoreactivity in the ipsilateral striatum in rats injected in the striatum with 6-OHDA (Barrachina et al., 2003). Significant increase in dendritic growth and branching of pyramidal neurons from the somatosensory cortex resulted in enlarging the surface area occupied by the neurons were observed in to Long Evans rats supplemented each day with CDP-choline from conception (maternal ingestion) to postnatal day 60 (Rema et al., 2008).

Marked and moderate improvement of cognitive functions was found in patients treated with α-GPC compared to the control one. Deterioration of cognitive functions was seen less often in the treated group than in the control group (Levin et al., 2009).

Treatment with α-GPC for at least 3 weeks was associated with neuroprotective effects and the ability to prevent BBB disruption in an animal model of pilocarpine-induced seizure (Lee et al., 2017). Administration of α-GPC, if immediately after seizure increased neuronal death, starting 3 weeks after administered seizure improved cognitive function through reduced neuronal death and BBB disruption, and increased neurogenesis in the hippocampus (Lee et al., 2018).

Phospholipid metabolites alterations were detected in postmortem AD brains (Nitsch et al., 1991). This supports the view that phospholipids turnover is elevated in neurodegenerative diseases (Nitsch et al., 1991). A mutual interaction between amyloid formation and membrane phospholipid breakdown has been suggested. The formation of amyloid peptide accelerates membrane breakdown, which is a typical feature of neuronal degeneration in acute and chronic disorders. Moreover, nerve cell loss and phospholipids breakdown may be involved in alterations of central cholinergic neurons. The supplementation of choline or CCPLs, could counter phospholipid breakdown by normalizing phosphatidylcholine, phosphatidyl-ethanolamine, and phosphatidylserine levels (Tayebati et al., 2013).

Based on the evidence that administration of CCPLs, increases ACh levels and modulates the phospholipid breakdown, the administration of CCPLs has been studied as a possible therapeutic approach to treat neurodegenerative disorders (Tayebati et al., 2013). Several studies have clarified the properties of CCPLs, in particular of α-GPC, on the neuronal alterations in animal models of cerebrovascular disease (Tomassoni et al., 2006, 2012; Tayebati et al., 2009, 2015). The beneficial effects of α-GPC may depend by an influence of the compound on brain phospholipids metabolism and/or by its documented activity of increasing free plasma choline levels (Gatti et al., 1992) and brain ACh bioavailability and release (Sigala et al., 1992). α-GPC provides both free choline and phospholipids for synthesizing ACh and could reconstitute the components of the nerves cell membrane. Moreover α-GPC has been found to increase ACh levels and release in rat hippocampus (Sigala et al., 1992; Amenta et al., 2006). These preclinical findings are associated with results of clinical trials in which CCPLs were proposed as potential pharmacotherapies for adult-onset dementia disorders. In particular, α-GPC countered the degeneration occurring in the brain areas involved in learning and memory of AD patients (Amenta et al., 2020).

More recently in a rat model of dual stress, the administration of α-GPC (400 mg/kg) for 7 days, countered the increase of stress hormones, reduced hearing loss, and prevented neuronal injury. This effect has been considered due to the increase of choline acetyltransferase (ChAT) and reduction of brain neuro-inflammation (Jeong Yu et al., 2022). Moreover, in the hippocampus α-GPC enhances brain-derived neurotrophic factor (BDNF) expression and protects the activity of immature cells in the hippocampus (Jeong Yu et al., 2022).

The CCPLs although show mainly a cholinergic profile and affect phospholipids biosynthesis, nervous tissue metabolism and neurotransmitter systems also may have a monoaminergic profile. Dopamine levels in brain areas increase after treatment for 7 days with α-GPC (150 mg/Kg/day) but not with CDP-choline (325 mg/Kg/day) whereas dopamine transporter expression was stimulated in frontal cortex and cerebellum by both CDP and α-GPC. α-GPC also increased serotonin levels in frontal cortex and striatum (Tayebati et al., 2013). CDP-choline in rats does not have effects in the forced swim test, but its primary metabolites showed opposing effects: cytidine has antidepressant-like actions, whereas choline has prodepressant-like actions (Carlezon et al., 2002). The results of this study are consistent with a clinical trial in which depression symptoms in mild to moderate stage AD patients, probably could benefit from stronger cholinergic stimulation induced by combined administration of donepezil and α-GPC (Carotenuto et al., 2022).

### Choline-containing phospholipids effects on astrocytes

In the NVU astrocytes play an important role in nutrition, support and protection of neurons and in the neuron-to-neuron signal transduction (Han et al., 2022). Astrocytes wraps the blood vessels with the end feet and mediates the transmission and the movement of molecules between neurons and blood vessels. Projections of astrocytes surround the neurons, can modulate the transmission and asset of synapses, and participate in the synaptic formation and neuronal differentiation (Stogsdill et al., 2017). It has been documented that estrogen released by cultured primary astrocytes, increased the number of newly formed synapses, and enhanced synaptic signal transmission (Fester and Rune, 2015). Cholesterol released by astrocytes induces synaptic formation and affects the number of synapses during the development of neurons (Mauch et al., 2001; Nakayama et al., 2003). Moreover, glial cells can deliver energy and nutrients for neurons, and release citrate, an important substance supplying neuronal energy during hypoglycemia (Meshitsuka and Aremu, 2008). Astrocytes represent a fundamental element in the BBB, a basic component of the NVU and a natural barrier for the brain to maintain the microvascular homeostasis. Astrocytes stretch out to wrap more than 90% of capillary endothelial cells and pericytes, interact with endothelial cells of brain microvasculature and maintain the BBB integrity (Thurgur and Pinteaux, 2018). Astrocytes with microglial cells monitor the brain microenvironment and constitutes the first line of the CNS defense (Li and Barres, 2018). In general, microglia and astrocytes are activated into two states: the neurotoxic phenotype (M1/A1) and the neuroprotection phenotype (M2/A2), corresponding to either the destructive and reparative functions in the NVU, respectively (Liu et al., 2020). Choline transporter-like 1 (CTL1) is expected to have a major role in the synthesis of phospholipids in the plasma membrane since it is functionally expressed in neurons and astrocytes in the CNS (Inazu et al., 2005; Fujita et al., 2006). Choline transporter 1, on the other hand, is expressed in cholinergic neurons and is connected to the production of acetylcholine (Okuda et al., 2000). Although the precise mechanisms are not yet understood, CTL1, which is expressed in astrocytes, is likely involved in astrocyte differentiation and proliferation (Machová et al., 2009). Membrane lipids like phosphatidic acid are probably transported from astrocytes to neurons either directly or indirectly since astrocytes are involved in the transit of many chemicals and proteins to neurons. It was found that astrocytes produce oleic acid, which was then hypothesized to be taken up by neurons for phospholipid production during neuronal development (Tabernero et al., 2001).

Choline-containing phospholipids modulate transglutaminase activity and expression in primary astrocyte cultures. Transglutaminase is an important Ca^2+^-dependent protein and a normal element of nervous systems during fetal stages of development. It plays a role in cell signal transduction, differentiation, and apoptosis. In astrocyte cultures supplemented with choline, CDP-choline, or α-GPC at 0.1 or 1 mM concentrations, confocal laser scanning microscopy analysis showed an increase of transglutaminase activity. Comparatively, α-GPC induced the most visible effects enhancing monodansyl-cadaverine fluorescence both in cytosol and in nuclei, supporting a possible active role played by α-GPC during differentiation processes. Western blot analysis showed that in 24 h 1 mM α-GPC and choline-treated astrocytes increased transglutaminase expression, whereas no effect was observed in 24 h 1 mM CDP-choline treated astrocytes (Bramanti et al., 2008a). Moreover, in primary astrocytes cell cultures at 14 and 35 days *in vitro*, CCPLs treatment for 24 h promoted a marked down-regulation of cyclin D1 expression, with reduced cyclin D1 expression in 1 mM α-GPC treated astrocytes. This suggests crucial role of CCPLs independent from ACh, on development and differentiation of astroglial cells *in vitro*, instead of on their maturation, proliferation, and development in culture (Bramanti et al., 2008b).

Another, similar, study in primary astrocytes cell cultures, showed a slight reduction of heme oxygenase-1 (HO-1) expression, a protein that plays a crucial role in oxidative stress processes, cell differentiation and apoptosis, in cell cultures of astrocytes treated with CDP-choline. On the contrary, ACh and choline induced a significant increase of HO-1 expression in 14 day *in vitro* astrocyte cultures (Bramanti et al., 2012). Results concerning p21 expression, a protein that inhibits the cell cycle, showed a significant increment with α-GPC treatment, while CDP-choline treatment caused a higher increase of p21 expression in 14 days *in vitro* astrocyte cultures. These data, suggest that CCPLs modulate HO-1 and p21 expression during astroglial cell differentiation and proliferation in culture and could be considered as a tool to study the induced effects of ischemia and hypoxia diseases (Bramanti et al., 2012). In astrocyte cultures treated with 50 μM (+) lipoic acid or (±)lipoic acid and/or 10 mM α-GPC for 24 h, induced an “upward modulation” in the expression of proliferating and differentiating biomarkers (Grasso et al., 2014).

In spontaneously hypertensive rats (SHR), a preclinical model of cerebrovascular disease associated with hypertension, a treatment for 4 weeks with an oral dose of 100 mg/kg/day of α-GPC decreased astrogliosis and restored expression of aquaporine-4, while galantamine (3 mg/kg/day) treatment countered to a greater extent than α-GPC neuronal alteration induced by hypertension (Tayebati et al., 2009). In the same animal model treatment with α-GPC countered neurons loss and glial reaction particularly in the CA1 subfield and in the dentate gyrus of the hippocampus of while phosphatidylcholine did not modify hypertension-dependent alterations in hippocampal microanatomy (Tomassoni et al., 2006).

In 32-weeks old SHR treated no significant changes in the size of perivascular astrocytes with a-GPC were observed compared to normotensive Wistar-Kyoto rats, while the expression of the BBB marker aquaporin-4 increased in SHR. This enhancement was countered by 4 weeks α-GPC (150 mg/kg/day) treatment. Endothelial markers and vascular adhesion molecules expression were not homogeneously affected by hypertension and α-GPC treatment in intracerebral vessels (Tayebati et al., 2015). This observation could explain data of clinical trials reporting an improvement of cognitive function in patients suffering from cerebrovascular disorders and treated with α-GPC (Amenta et al., 2020).

### Choline-containing phospholipids effects on microglia

As brain-resident immune cells, microglia monitor the microenvironment and are ubiquitously distributed throughout the CNS. Microglial cells have been designated as branched, tissue-resident macrophages more abundant in the brain, and represent approximately the 20% of the total glial cells (Wang et al., 2022).

M1–M2 classification, which was applied to peripheral macrophages, divides the microglial activation state into classical activation (M1) or alternative activation (M2) (Dubbelaar et al., 2018). According to the morphology and functions, microglial cells are divided into three categories: M0, M1, and M2. The M0 phenotype, known as the “resting” microglia phenotype, represents microglia that are highly active in their presumed resting state. It is involved in monitoring the presence of pathogens in the local environment and the changes in extracellular concentrations of constitutively expressed neurochemicals (Nimmerjahn et al., 2005). The cells sense the microenvironment by interacting with neurons, blood vessels, ependymal cells, and astrocytes. The M1 phenotype is characterized by the production of pro-inflammatory cytokines (such as TNF-α, IL-6, and IL-1β), chemokines and reactive oxygen species (ROS), leading to an acute immune response. On the contrary, M2 phenotype is characterized by the production of anti-inflammatory cytokines (IL-4 and IL-13), involved in the tissue repair, wound healing, wastes removal, and in the renovation of brain homeostasis (Cherry et al., 2014). The administration of CCPLs had no impact on the blood levels of the microglial marker ionized calcium-binding adaptor molecule 1 (Iba1), possibly protecting the microglia’s crucial functional role in the resolution of local inflammation, the removal of cellular debris, and the provision of protective factors to lessen cell injury in the ischemic brain. Some investigations have shown that serum levels of neuron-specific biomarkers are important and clinically useful indicators of the effectiveness of treating pathologies, and they can be used for further research into the pathophysiology of stroke and the molecular mechanisms underlying nootropic-mediated neuroprotection (Kuryata et al., 2021). ROS and pro-inflammatory cytokines are released by activated microglia and are toxic to nearby neurons, astrocytes, and oligodendrocytes. However, microglia have the capacity to reduce astrocyte reactivity, clear cellular debris, and produce protective substances that can lessen oligodendrocyte injury and/or demyelination as well as local inflammation (Xu et al., 2020). The phagocytosis of active microglia requires the calcium binding protein Iba1, which is specific to macrophages and microglia (Sasaki et al., 2001). Recently, it has been shown that GFAP and Iba1 are two additional biomarkers for neuroinflammation following mild traumatic brain injury (Lafrenaye et al., 2020). CCPLs-based therapeutic strategy to target for demyelination and increasing long-term neurological function (Jayaraj et al., 2019). During systemic inflammation, activated microglial units behave similarly to macrophages and show the potential to phagocytose harmful cellular debris (Lee et al., 2019). Administration of citicoline to the hippocampus significantly decreased microglial activity. In order to more carefully assess the polarization states of microglia, studied at the expression of M1 (IL-6) producers in Iba1-positive microglia. This revealed that citicoline administration may inhibit M1 polarization (Kim J. H. et al., 2018).

Choline-containing phospholipids interact with microglia and exert anti-inflammatory properties in animal models of cerebral disease, while in normal conditions these molecules did not affect the expression of inflammation markers, both inflammation cytokines and endothelial adhesion molecules, in different cerebral areas.

The toll-like receptor (TLR) stimulation enhances choline uptake by macrophages and microglia by the induction of the choline transporter CTL1. Inhibition of CTL1 expression or choline phosphorylation attenuated NLRP3 inflammasome activation and IL-1β and IL-18 production in stimulated macrophages. Correspondingly, choline kinase inhibitors ameliorated acute and chronic models of IL-1β-dependent inflammation (Sanchez-Lopez et al., 2019).

In a rodent model of systemic inflammation induced by i.p. treatment of lipopolysaccharide (LPS), the administration of a special diet enriched with phosphatidylcholine (1% for 5 days before the administration of LPS and thereafter during the 7-day observation period) induced a reduction in the plasma TNF-α and hippocampal NOx changes, prevented the decrease of neurogenesis and the microglial activation (Tokés et al., 2011).

Phosphatidylcholine treatment significantly attenuated docetaxel-induced peripheral neurotoxicity. Phosphatidylcholine decreased oxidative stress in sciatic nerve ameliorated docetaxel-induced neuronal damage and microglial activation in the sciatic nerve and spinal cord (Kim S. T. et al., 2018). The prevention of microglia activation represents a neuroprotective strategy in a rat model of insulin-induced hypoglycemia. In the brain of these rats, the activated microglia represent the M1 polarization and that CDP-choline injections (500 mg/kg, i.p.), may reduce M1 polarization as well as microglial activation following hypoglycemia (Kim J. H. et al., 2018).

In a rodent model of oxaliplatin-induced neuropathy phosphatidylcholine attenuated oxidative stress by increasing antioxidant levels and immunohistochemical evaluation, indicated that phosphatidylcholine administration ameliorated microglial activation, suggesting a therapeutic effect against oxaliplatin-induced peripheral neuropathy due to its antioxidant property (Kim S. T. et al., 2015). Surprisingly, CDP-Choline treatment enhanced seizure-induced neuronal death and microglial activation in the hippocampus of an animal model of pilocarpine-induced epilepsy. CDP-choline administration after seizure-induction increased immunoglobulin leakage *via* BBB disruption indicating that the compound was not able to modulate neuronal death, BBB disruption or microglial activation (Kim S. T. et al., 2015).

α-GPC (0.07 mg/ml) administered in water decreased the deposition of transthyretin (TTR), an amyloidogenic protein, and led to neuroinflammation in senescence-accelerated mouse prone 8 mice. These effects suggest that α-GPC may be a useful compound in anti-aging functional food development (Matsubara, et al., 2018).

In spontaneously hypertensive rats, similarly to the reduction of astrogliosis, α-GPC induced a reduction of microglial activation (Tayebati et al., 2015), while in the same animal model vascular adhesion molecules and endothelial markers expression were not homogeneously modified by hypertension or α-GPC treatment (Tayebati et al., 2015).

The nutritional importance of α-GPC in combination with docosahexaenoic acid (DHA) plus triglyceride (TG) was studied. The date showed that the α-GPC in combination with DHA + TG is more neurodevelopmentally effective than DHA + TG or DHA + TG + phosphocholine or DHA + phosphocholine (Tayebati, 2018).

### Choline-containing phospholipids effects on endothelial cells and pericytes

Metabolites and neurotoxic substances produced in the brain are cleared through the action of ATP-binding cassette transporters, such as P-glycoprotein, which are found at the luminal membrane of endothelial cells (Okura et al., 2008; Shimomura et al., 2013). Under physiological circumstances, choline has a positive charge and is a cationic chemical. Therefore, it is anticipated that cationic medicines will competitively limit choline transporter. Indeed, the uptake of several cationic medications (such as clonidine, desipramine, diphenhydramine, quinidine, quinine, and verapamil) hindered choline uptake in human brain microvascular endothelial cells in a concentration-dependent manner (Iwao et al., 2016). Spherical, solitary cells called pericytes are near endothelial cells and they support endothelial cells by anatomical contact.

The effects of CDP-choline on the permeability and expression of tight junction proteins in endothelial cells were tested in an *in vitro* model of human umbilical vein endothelial cells (HUVECs) of hypoxia/aglycemia conditions. In this model, the increased permeability of HUVECs accompanied with down-regulation of zonula occludens-1 (ZO-1) and occluding. Citicoline decreased the permeability with an increased expression of the TJPs, demonstrating that citicoline restores the barrier functions of endothelial cells (Ma et al., 2013). The treatment of CDP-choline (500 mg/kg) in an animal model of cerebral infarct, enhanced cell proliferation, vasculogenesis and presynaptic proteins expression, and reduced astrogliosis levels in the peri-infarct area of the ischemic stroke (Gutiérrez-Fernández et al., 2012a,b). In acute ischemic stroke patients, the increase levels of the in circulating endothelial progenitor cells (EPCs) is associated with a better outcome. A combination of CDP-choline (2,000 mg/day) and recombinant tissue-plasminogen activator (rt-PA) given in acute ischemic stroke and at 6 weeks was able to increase EPC levels and caused protective effects (Sobrino et al., 2011).

The main preclinical observation of possible effects of CCPLs on *in vitro* and *in vivo* models are summarized in Table 2.

**TABLE 2:** Effects of choline-containing phospholipids (CCPLs) in different elements of neurovascular unit (NVU) evaluated in different *in vitro* and *in vivo* models.

*In vitro* or *in vivo* model	Evidence	References
**Main results for neurons**		

Neuronal cell cultures	Phosphatidylcholine could play a signalling role during neuronal differentiation	Paoletti et al., 2011
Primary cultured neurons treated with Aβ1-42-induced damage	Phosphatidylcholine prevented neuronal death	Ko et al., 2016
Aged mice	Phosphatidylcholine had no effect on hippocampal dendritic spine density compared to control, but improved learning and memory	Muma and Rowell, 1988
Wistar rats with ischemia/reperfusion (I/R)-induced insult	Phosphatidylcholine has a possible neuroprotective effect partly through its antioxidant action	Aabdallah and Eid, 2004
Aging rats	Dietary CDP-choline supplementation can protect impairment in hippocampal-dependent long-term memory.	Teather and Wurtman, 2003
Long Evans rats	CDP-choline significant increases in neurite length, branch points and total area occupied by the neurons were observed	Rema et al., 2008
Human dopaminergic SH-SY5Y neuroblastoma cells	CDP-choline reduces the cytotoxic effect of 6-hydroxydopamine (6-OHDA)	Barrachina et al., 2003
Rats	α-GPC increase acetylcholine level, facilitates learning and memory, improves brain transduction mechanisms	Traini et al., 2013
Rats	α-GPC increase dopamine levels, whereas dopamine transporter expression was stimulated by both CDP and α-GPC in brain areas	Tayebati et al., 2013
Spontaneously hypertensive rats (SHR) as a model of cerebrovascular disease	α-GPC prevent neuronal alterations	Tayebati et al., 2009, 2015; Tomassoni et al., 2006, 2012
Pilocarpine-induced seizure in rat	α-GPC, starting 3 weeks after seizure improved cognitive	Lee et al., 2018
Rat model of dual stress	α-GPC countered the increase of stress hormones, reduced hearing loss, and prevented neuronal injury	Jeong Yu et al., 2022

**Main results for astrocytes**		

Primary astrocyte cultures	Active role played by CCPLs., particularly a-GPC throughout differentiation processes	Bramanti et al., 2008a,b, 2012; Grasso et al., 2014
Spontaneously hypertensive rats (SHR) as a model of cerebrovascular disease	α-GPC reduced astrogliosis and the expression of aquaporin-4	Tayebati et al., 2009, 2015; Tomassoni et al., 2006
Rat with middle cerebral artery occlusion as a model of stroke	CDP-choline reduced glial fibrillary acidic protein levels in the peri-infarct area of the ischemic stroke	Gutiérrez-Fernández et al., 2012a,b

**Main results for microglia**		

Rat model of on oxaliplatin-induced neuropathy	CDP-choline administration ameliorated microglial activation	Kim S. T. et al., 2015
pilocarpine-induced epilepsy in rat	CDP-Choline treatment enhanced seizure-induced neuronal death and microglial activation	Kim J. H. et al., 2015
Rat with docetaxel-induced peripheral neurotoxicity	CDP choline significantly decreased microglial activation and M1 polarization in rat hippocampus	Kim S. T. et al., 2018
Senescence-accelerated mouse prone 8 (SAMP-8)	α-GPC protect the brain by reducing TTR deposition and preventing neuroinflammation.	Matsubara et al., 2018
Spontaneously hypertensive rats (SHR) as a model of cerebrovascular disease	α-GPC induce a reduction of microglial activation	Tayebati et al., 2015

**Main results for endothelial cells and pericytes**		

Human umbilical vein endothelial cells (HUVECs) under hypoxia/aglycemia conditions	CDP-choline decreased the permeability with an increased expression of the tight junction proteins	Ma et al., 2013
Rat with middle cerebral artery occlusion as a model of stroke	CDP-choline increased cell proliferation, and vasculogenesis	Gutiérrez-Fernández et al., 2012a,b
Spontaneously hypertensive rats (SHR) as a model of cerebrovascular disease	α-GPC affect endothelial markers and vascular adhesion molecules expression ICAM-1, VCAM-1, and PECAM-1	Tayebati et al., 2015

## Clinical activity of choline-containing phospholipids

A natural question after the above description of the role of CCPLs is if and to what extent this class of molecules with a large and documented NVU activity has shown an efficacy in brain disorders characterized by cerebrovascular impairment. CCPLs play an important role in cellular metabolism and are relevant components of various cell membranes (i.e., cellular, mitochondria, endoplasmatic reticulum, Golgi apparatus, peroxisomes, and lysosomes). Choline is a crucial nutrient with a complex role in the body, is necessary for the biosynthesis of the neurotransmitter ACh and is also the major dietary source of methyl groups *via* the synthesis of *S*-adenosylmethionine (Tayebati and Amenta, 2013).

The first approach to restoring deficient cholinergic neurotransmission and alleviating cognitive impairment in dementia disorders was cholinergic precursor-loading therapy. Taking into account the controversial nature of the existence of a direct role of choline on ACh release and given that an increase of brain free choline does not always imply an increase of brain ACh (Tayebati and Amenta, 2013), CCPLs were then more largely investigated. Cholinergic neurons probably have a particular avidity for choline, and it has been hypothesized that when the provision of choline is insufficient, neurons obtain it by hydrolyzing membrane phospholipids. This mechanism, known as auto cannibalism, may render cholinergic neurons more susceptible to injury and may contribute to cholinergic neuron degeneration (Wurtman et al., 1990).

The CCPLs proposed for the treatment of age-related dementia disorders include lecithin, CDP-choline and α-GPC. The main evidence of their activity is reviewed below and summarized in Table 3.

**TABLE 3:** Evidence of the effects of choline-containing phospholipids (CCPLs) in clinical studies.

Disease	Types of study and patients	Evidence	References
**Phosphatidylcholine**			

Dementia and cognitive decline	265 with Alzheimer’s disease, 21 with Parkinsonian dementia and 90 with subjective memory problems in randomized trials	Evidence doesn’t support the use of lecithin in the treatment of patients with dementia	Higgins and Flicker, 2003
	2,497 in a population study	Higher phosphatidylcholine intake was associated with lower risk of incident dementia and better cognitive performance in men	Ylilauri et al., 2019

**CDP-choline**			

Acute ischemic stroke	4,281 patients in randomized controlled trials	Little to no difference compared controls regarding all-cause mortality, disability or dependence in daily activities, severe adverse events, functional recovery and neurological function	Martí-Carvajal et al., 2020
Chronic cerebral disorder in elderly and dementia	1,307 in clinical trials	Evidence that CDP-choline has a positive effect on memory and behaviour	Fioravanti and Yanagi, 2005
	736 in observational studies	Reduction of cognitive dysfunction Reduction of disturbances of visual/spatial Positive effects on the emotional Decreasing the level of depression	Mashin et al., 2017
	174 Outpatients in retrospective case-control study (CDP-Choline + Rivastigmine)	Effectiveness of combined administration versus the Rivastigmine alone, mainly in slowing disease progression and consequently in disease management, of Alzheimer’s disease (AD) and in mixed dementia (MD)	Castagna et al., 2016
	170 Retrospective multi-centric case-control study	After 12 months, triple therapy with citicoline, memantine, and AChEI was more effective than memantine and AChEI without citicoline in maintaining the MMSE total score	Castagna et al., 2021a
	104 in retrospective case-control study	Absence of a statistically significant difference between case and control groups for the MMSE total scores	Castagna et al., 2021b

**Choline alphoscerate**			

Acute cerebrovascular disease	4,315 in 11 trials on dementia disorders of neurodegenerative or vascular origin and in cerebrovascular disease	Choline alphoscerate provided some modest symptomatic relief primarily on memory and attention	Parnetti et al., 2007
AD associated with cerebrovascular injury	113 Participants to the randomized, placebo-controlled, double-blind ASCOMALVA trial	Findings suggest that the combination of choline alphoscerate with a ChE-I may prolong/increase the effectiveness of cholinergic therapies in AD with concomitant ischemic cerebrovascular injury	Amenta et al., 2014
	56 Participants to the randomized, placebo-controlled, double-blind ASCOMALVA trial	Addition of choline alphoscerate to standard treatment with the cholinesterase inhibitor donepezil counters to some extent the loss in volume occurring in some brain areas of AD patients	Traini et al., 2020
Mild cognitive impairment	50 Patients	Psychometric measures showed a significant improvement	Gavrilova et al., 2018
Ischemic and hemorrhagic stroke	277 Patients in 6 trials	Regress in neurological deficit, better recovery in cognitive functions and functional status	Traini et al., 2013
chronic brain ischemia and moderate cognitive impairment.	25 Patients, 16 women, and 9 men	significant positive effect on patient’s condition including cognitive function	Pizova, 2014
Parkinson’s disease with cognitive disorders	40 Patients (main group) 20 patients (control group)	Marked and moderate improvement of cognitive functions was found in patients of the main group compared to the control one	Levin et al., 2009
vascular cognitive impairment (VCI) due to cerebral small vessel disease (SVD).	62 Patients, pilot, single-center (university hospital), double-blinded, randomized clinical trial (Choline Alphoscerate and Nimodipine)	Combined choline alphoscerate-nimodipine treatment showed no significant effect	Salvadori et al., 2021

### Phosphatidylcholine

Phosphatidylcholine is a CCPLs representing the key font of choline. Phosphatidylcholine increases serum choline levels more effectively than orally administered choline (Wurtman et al., 1990). Apparently, phosphatidylcholine may accelerate ACh synthesis in the brain through enhanced availability of choline. Lecithin has been tried out in the treatment of dementia, alone or in combination with an acetylcholinesterase (AChE) inhibitor. Phosphatidylcholine is mainly available as a nutraceutical. The role of this compound in cognitive impairment and dementias has been the focus of a Cochrane review in 2003 (Higgins and Flicker, 2003). No trials reported any clear clinical benefit of phosphatidylcholine for AD or Parkinson’s dementia. Only scarce clinical trials have led to data to meta-analyses. The only statistically significant result was in favors of placebo for adverse events, based on one single trial. Relevant results in favor of phosphatidylcholine were gained in a clinical study of subjects with individual problems of cognition (Higgins and Flicker, 2003). More recently the associations of dietary choline intake with the risk of incident dementia and with cognitive performance in middle-aged and older men were evaluated. Total choline and phosphatidylcholine intakes were associated with better performance in cognitive tests and higher phosphatidylcholine intake was associated with lower risk of incident dementia and better cognitive performance in men (Ylilauri et al., 2019).

### Cytidine 5′-diphosphocholine

CDP-choline is a CCPLs composed by choline and cytidine, joint together by a diphosphate bridge (Figure 1C). Citicoline is the international non-proprietary name of CDP-choline. The functions of CDP-choline include the restoration of the neural membrane through the synthesis of phosphatidylcholine, the reduction of stored fat responsible for the cognitive decline, and the increase of ACh levels. In post-stroke patients a neuroprotective effect of CDP-choline, contributing to an improvement in attention tasks, executive function, temporal orientation, along with an ability, although in an experimental setting, to improve the neural repair has been reported.

Several studies have demonstrated positive effects of the compound on cognition, but other studies failed to confirm the results. In view of these discrepancies, additional clinical studies are necessary to confirm the potential benefits of citicoline in the treatment of adult-onset dementia disorders (Amenta et al., 2020).

### Choline alphoscerate

α-GPC has been on the pharmaceutical market since 1987, however, its popularity has declined since the introduction of cholinesterase inhibitors. The last 10 years have seen a renewed attention on this compound, with preclinical studies, clinical investigations and review articles published in the literature (Traini et al., 2013).

The majority of clinical studies available on the effect of α-GPC on cognitive function in neurodegenerative and cerebrovascular disorders were detailed in two review articles as showed in Parnetti et al. (2007) and Traini et al. (2013). Administration of α-GPC improved cognitive functions, as well as affective and somatic symptoms (fatigue, vertigo). The effects of α-GPC were greater than those of placebo and of the same extent or superior to those of reference compounds (Amenta et al., 2001). Studied in rat models showed that the association of α-GPC with AChE inhibitors enhance the effects of both drugs on cholinergic neurotransmission (Amenta et al., 2006). Based on this evidence an independent clinical trial: “Effect of association between a cholinesterase inhibitor and α-GPC on cognitive deficits in AD associated with cerebrovascular impairment” (ASCOMALVA) was performed in Italy. In agreement with literature data, in patients selected to the reference treatment group (donepezil + placebo), a slight time-dependent worsening of Mini Mental State Examination (MMSE) and the Alzheimer’s Disease Assessment Scale–Cognitive Subscale (ADAS-cog) scores was found. In the active treatment group, the administration of donepezil+ α-GPC countered the decline of MMSE and ADAS-cog scores. The effect of the association on psychometric tests was statistically significant after 12 months of treatment (Amenta et al., 2014). The combination of donepezil plus α-GPC was more effective than donepezil alone in countering symptoms of apathy in AD. This suggests that the availability in the brain of a higher amount of acetylcholine may affect apathy in AD subjects with spared executive functions (Rea et al., 2015).

A supplementary contribution of the ASCOMALVA trial was the study of the influence of the combine treatment of α-GPC and donepezil on brain atrophy in AD. Cerebral atrophy is a common feature of neurodegenerative disorders. This pathology includes a loss of gyri and sulci in the temporal lobe and parietal lobe, and in parts of the frontal cortex and of the cingulate gyrus. Patients enrolled in the ASCOMALVA trial underwent yearly Magnetic Resonance Imaging (MRI) analysis for diagnostic purposes. In 56 patients who achieved 3 years of therapy, brain MRI were examined by voxel morphometry techniques. At the end of 3 years of treatment, in patients enrolled in the active group of donepezil plus α-GPC, a reduction of the volume loss of the grey matter (with a concomitant increase of the volume of the ventriculi and cerebrospinal fluid space) was observed, compared to the reference group (donepezil alone). Frontal and temporal lobes, hippocampus, amygdala and basal ganglia represented the areas in which brain atrophy was more sensitive to combination treatment. No significant differences were noticeable in other areas, between the two groups. Morphological data were confirmed by neuropsychological assessment performed alongside the trial (Traini et al., 2020).

In conclusion, a cholinergic precursor-loading strategy with α-GPC in combination with donepezil counters to some extent the atrophy occurring in some brain areas of AD patients. In these patients, the parallel observation of an improvement in cognitive and functional tests suggests that morphological changes observed could have functional relevance (Traini et al., 2020).

## Conclusion

**FIGURE 3:**
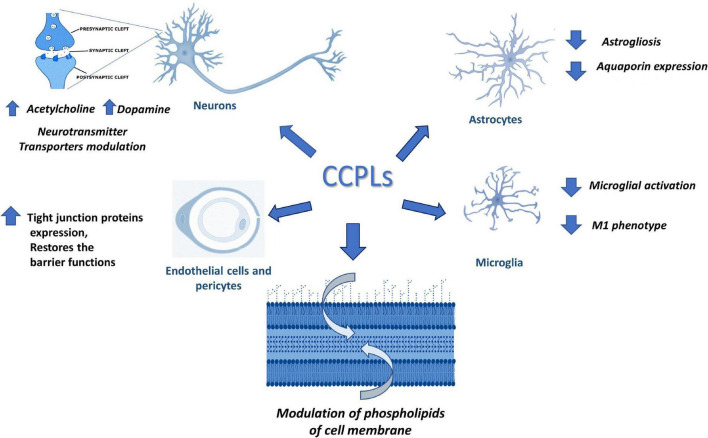
Possible activity of choline containing phospholipids (CCPLs) on different elements of Neurovascular Unit or in cell membrane phospholipids metabolism.

This manuscript has reviewed the role of CCPLs on the function of the different components of the NVU. CCPLs promote metabolism in different elements of the NVU by increasing the synthesis of neurotransmitters, or by incorporation into the membrane phospholipid structure, improving synthesis of phospholipids and mitochondrial metabolism, as shown in Figure 3.

A comparative analysis on the results obtained with animal models and in clinical trials revealed a discrepancy between quite positive results observed in preclinical studies and the limited positive outcomes reported in clinical trials. The value of animal research in drug development is increasingly questionable primarily in case of typical human diseases such as neurodegenerative disorders. For instance, a relevant lack of success in AD drug development over the past two decades was observed (Kim et al., 2022), whereas preclinical studies reported quite encouraging results. Several factors can contribute to these inconsistencies (Mehta et al., 2017). One is the inadequacy in the selection of animal models not always done with a clear and specific translational rationale. Another reason could be the lack of experiments conducted according to the best practices and mimicking the course of a human disease. We should also consider that the use of animal models such as the rodents with a short lifespan compared with the long time necessary for the development of symptoms of a typical neurodegenerative disorder represents a potential obstacle for the translatability of animal data to humans. On the other hand, treatment starting in advanced stages of a disease is difficult to bring positive results. Hence, the use of suitable animal models and the treatment of diseases for enough time at a not too advanced stage may represent a reasonable approach to give a true value of evidence coming from animal studies.

Despite the large evidence about the biochemistry of these molecules and the role in modulating the activity of the NVU, clinical studies on the activity of CCPLs in pathologies have suggested a dysfunction of the NVU (adult-onset dementia disorders of vascular origin) are limited. Phosphatidylcholine was the first cholinergic precursor molecule used, but it did not demonstrate clear clinical benefits. The same is not true for other phospholipids, CDP-choline and α-GPC, involved in choline biosynthetic pathways. For these an uncertain improvement of cognitive dysfunction in neurodegenerative and vascular dementia is documented. Positive results obtained with selected cholinergic precursors cannot be generalized due to the small numbers of patients studied in appropriate clinical trials. However, they probably would justify reconsideration of the most promising molecules in larger carefully controlled studies. This can also contribute to better define the role of the NVU in the pathophysiology of brain disorders characterized by vascular impairment.

## Author contributions

PR, DT, ET, and GN conducted critical analysis of the literature on the topic of manuscript. DT and FA designed the review. PR, DT, and ET wrote the draft of the manuscript. PR, DT, ET, GN, and FA revised the ultimate version of the manuscript. All authors contributed to the article and approved the submitted version.
